# Polypeptide Chain Growth Mechanisms and Secondary Structure Formation in Glycine Gas-Phase Deposition on Silica Surfaces

**DOI:** 10.1021/acs.jpcb.2c07382

**Published:** 2023-01-13

**Authors:** Ola El Samrout, Gloria Berlier, Jean-François Lambert, Gianmario Martra

**Affiliations:** † Department of Chemistry and NIS Centre, University of Torino, Via P. Giuria 7, 10125Torino, Italy; ‡ Laboratoire de Réactivité de Surface, LRS (UMR 7197 CNRS), Sorbonne Université, Place Jussieu, 75005Paris, France

## Abstract

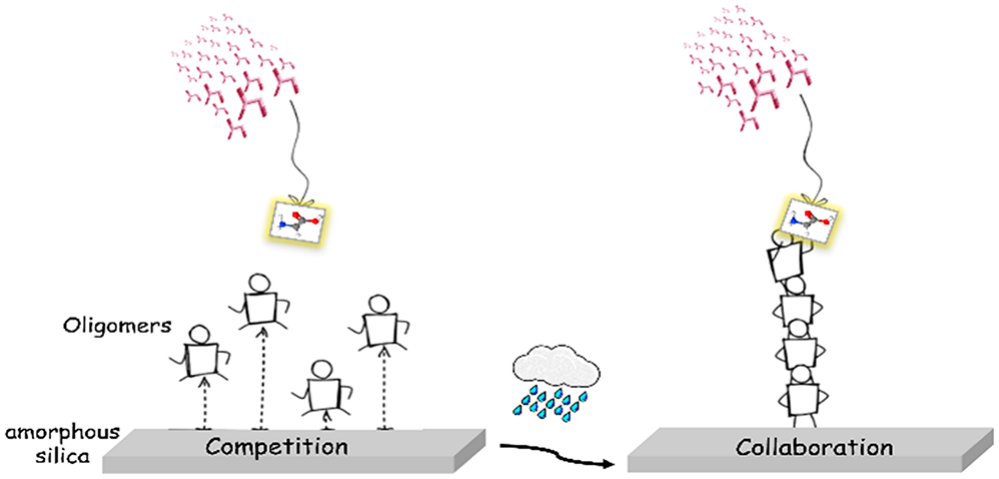

Peptide formation by amino acids condensation represents a crucial reaction in the quest of the origins of life as well as in synthetic chemistry. However, it is still poorly understood in terms of efficiency and reaction mechanism. In the present work, peptide formation has been investigated through thermal condensation of gas-phase glycine in fluctuating silica environments as a model of prebiotic environments. In-situ IR spectroscopy measurements under a controlled atmosphere reveal that a humidity fluctuating system subjected to both temperature and water activity variations results in the formation of more abundant peptides compared to a dehydrated system subjected only to temperature fluctuations cycles. A model is proposed in which hydration steps result in the hydrolysis and redistribution of the oligomers formed during previous deposition in dry conditions. This results in the formation of self-assembled aggregates with well-defined secondary structures (especially β-sheets). Upon further monomers feeding, structural elements are conserved in newly growing chains, with indications of templated polymerization. The structural dynamics of peptides were also evaluated. Rigid self-assembled structures with a high resistance to further wetting/drying cycles and inaccessibility to isotopic exchange were present in the humidity fluctuating system compared to more flexible structures in the dehydrated system. The resistance and growth of self-assembled structures were also investigated for an extended duration of Gly deposition using isotope labeling.

## Introduction

Among the different types of biomolecules reactions on mineral surfaces, amide/peptide bond formation through the condensation of amino acids is of high relevance due to its direct applications in various sectors ranging from bio-/nanotechnology and drug delivery^1^ to the quest of the origins of life.^2^ A lot of research works have been devoted to study the mechanism, rate of amino acids polymerization reaction, and the self-assembly of resulting peptides. In particular, in the field of prebiotic chemistry, systems consisting of a solid (mineral) surface, water, and amino acid (AA) monomers were critically analyzed and evaluated for this reaction, including solid–gas, solid–liquid, solid–liquid–solid (mineral + aqueous solution + ice), and solid–liquid–gas, and so-called “fluctuating systems” undergoing wetting/drying (WD) cycles. However, the conclusions of these studies are most often purely empirical due to the lack of clear experimental data to support specific mechanisms for condensation and polymerization reactions. Each of these systems may be considered to represent the idealization of a particular prebiotic environment, and in particular, the solid–gas system is representative of a dehydrated lagoon floor produced after the evaporation of the liquid phase.^3^ One simplified experimental model to carry out the adsorption and polymerization of amino acids from the gas phase is by using chemical vapor deposition (CVD), a solvent-free method that can be conducted in mild reaction conditions without the use of activating agents.^4^ The fluctuating solid–liquid–gas system with wetting–drying cycles simulates in the lab natural prebiotic variations of the experimental conditions that may have occurred on the prebiotic earth such as daily fluctuations of temperatures and seasonal fluctuations of humidity where hydration (flooding, tidal variation, and rainstorms) and dehydration (evaporation or exposure to the sun under prebiotic conditions) take place in a cyclic manner.^2,5^

It has been long ago suggested by Lahav et al.^3^ that such fluctuating systems with both water content and temperature fluctuations should constitute the most favorable and geologically relevant settings for prebiotic condensation reactions. The argument, which has been rephrased several times,^6^ is that the drying phase drives the condensation reaction by removing water, a product of the condensation reaction, while the wet phase promotes the diffusion of reactants on the solid surface for a better reaction efficiency. Indeed, polymerization of various amino acids and their mixtures on several mineral phases subjected to WD cycles has been observed experimentally to yield oligopeptides up to at least the pentamers.^7,8^ In particular, an experimental study dealing with the thermal condensation of glycine (Gly) in fluctuating clay environments^5^ showed that a system subjected to cyclic variations in both temperature and water content resulted in a higher yield of oligopeptides as compared with one undergoing only temperature fluctuations (in this case, applied on wet systems).

Apart from peptide formation, the biomolecules–mineral–water interface is also important for prebiotic chemistry as it can promote the self-assembly of biomolecules, a possible step toward the formation of more complex structures. The study of self-assembling peptides has undergone a significant growth since the early 1990s due to their relevance in a large number of areas such as tissue engineering, biomedicine, synthetic biology, and beyond.^9,10^ Many studies have also demonstrated the self-assembly of peptide systems in the synthesis of nanomaterials. For instance, polyGly chains about 16 units long can form self-assembled aggregates containing both helical and β-sheet secondary structures on oxide surfaces including TiO_2_ and amorphous SiO_2_, upon contact with water vapor after CVD deposition.^4^ The natural abundance and low cost of silica stimulate current efforts to find catalytic routes for condensation and polymerization reaction on its surfaces.^4,11^

A previous paper has dealt with the initial steps of glycine polymerization on the silica surface upon deposition from the gas phase.^12^ In the present work, we aim to study the effect of different environmental conditions on the extent and rate of the polymerization reaction and especially on the self-assembly of three systems composed of amorphous silica (AX), a model of one of the most common mineral surfaces available on primitive Earth and other extraterrestrial bodies, glycine (Gly), the most prevalent amino acid in the earliest proteins, and water, the predominant solvent on the early Earth’s surface. Namely, the three systems were subjected separately to (a) temperature fluctuations (TF) during chemical vapor deposition (CVD), (b) both TF and humidity fluctuations (HF) cycles, and (c) TF and HF cycles for an extended time of CVD (35 h) while using a silica surface subjected to conditions of high water activity before the start of the polymerization. The types and relative concentrations of the secondary structures formed during the intermediate cycles of polymerization were assessed. Furthermore, the structural dynamics of the polypeptides were studied for the three systems and related to the kinetics of H/D exchange.

Infrared (IR) spectroscopy was the major technique adopted in this work for in-situ characterization of the growing peptides chains throughout glycine deposition. IR is highly sensitive, compared to other spectroscopic methods, to the H-bonding state and conformation of the different peptide groups. It has proven to be a useful and powerful technique in the study of protein structures, providing not only qualitative information about the presence of functional groups in organic compounds but also a quantitative estimation of the protein secondary structures yielding important structural and dynamical information about the peptides.^13^

While multinuclear solid-state NMR has been successfully used to amino acids transformations on silica,^14,15^ it can only be applied after completion of the whole reaction, and it is difficult to avoid re-exposure to air. In the same way, circular dichroism (CD), also commonly used to study the secondary structures of proteins, requires a prior extraction of the peptides from the surface to study their secondary structures. IR spectroscopy, on the other hand, does not require extensive sample preparation or extraction and can be applied without exposure to air.^16^ Moreover, the use of isotope labeling during IR spectroscopy measurements on biomolecules may help identify subtle conformational changes in the peptide secondary structures and probe specific local structures dynamics in the system, using deuterated water (D_2_O) instead of light water (H_2_O) or amino acids labeled with ^13^C, ^15^N, etc.^17^

## Experimental Section

### Materials

A pyrogenic amorphous silica Aerosil OX 50 (AX) (SiO_2_ content ≥99.8 wt %, specific surface area 50 m^2^ g^–1^) provided by EVONIK was used. Natural abundance glycine and ^13^C (99 atom % ^13^C) as well as ^15^N-enriched (98+ atom% ^15^N) glycine were purchased from Sigma-Aldrich. Deuterated water (D_2_O, 99.90 atom % D), a high-purity product obtained from Sigma-Aldrich, and Milli-Q water (Millipore system) were subjected to several freeze–pump–thaw cycles before admitting them in the IR cell through the vacuum line.

### Dehydration of the Silica Surface

Silica pellets were outgassed in a vacuum in the IR cell at room temperature (rt) or 160 °C (designated later as AX_rt_ and AX_160_, respectively) to remove the physisorbed water molecules and reach a good surface dehydration level.

### Gly Adsorption from the Gas Phase under Temperature Fluctuations (CVD with TF)

After dehydration of the silica surface under vacuum, glycine (Gly) sublimation and polymerization on the silica surface were performed at 160 °C using the chemical vapor deposition (CVD) method described by Martra et al.^4^ Briefly, the pretreated pellet of silica, held in a gold frame, was placed next to a pellet of Gly within a section of the IR cell acting as a reactor; a tubular furnace was placed around this part to heat it to 160 °C under a static vacuum. The temperature was measured and controlled using a thermocouple placed on the external part of the cell during the reaction. The IR cell was connected to a liquid nitrogen trap to remove any water generated during the reaction from the system. For IR spectra collection, the temperature was cooled to room temperature (rt), and the silica pellet was moved to the part of the cell equipped with CaF_2_ windows. The sublimation of Gly on the silica surface was performed in successive steps as follows: (i) heating the experimental device (silica and Gly pellets) from room temperature to 160 °C for 2.5 h; (ii) cooling from 160 °C to room temperature (rt) for 14 h; and (iii) repetition of the steps i and ii *n* times (*n* = number of temperature fluctuation cycles). After each cycle, the sample was subjected to in-situ IR measurements to follow the chemical transformation of the amino acid on the surface.

### Hydration Fluctuations (HF) Cycle Procedure, and H/D Isotopic Exchange

Before water vapor exposure, the sample, held in a gold frame in the IR cell, was outgassed under vacuum at rt until the residual pressure (<10^–3^ mbar) was achieved. Water vapor under saturating pressure was then admitted at rt to the surface of the sample which was kept under water vapor while collecting in-situ IR spectra until invariance of spectra (ca. 15 min). After that, the water vapor was removed by outgassing at rt until invariance of spectra. For H/D isotopic exchange, the same procedure was followed using D_2_O vapor for many cycles until invariance of in-situ IR spectra.

### Wetting/Drying (WD) Cycles with Liquid Water

After the experiments involving Gly adsorption and H_2_O and D_2_O vapor exposures, the sample pellet was removed from the cell, manually ground in an agate mortar, and then suspended in 0.5 mL of Milli-Q water. The suspension was shaken by a vortex mixer for 15 min and then centrifuged for 10 min at 10000 rpm. After removal of the supernatant, the solid was subjected to a second washing. The two aliquots of the aqueous solution obtained were mixed for analysis by high-resolution mass spectrometry. The same procedure of wetting/drying cycles was applied to a bare silica before any Gly deposition. After washing, the solid samples were dried under nitrogen flow, then pelletized again, and introduced in the IR cell for subsequent IR measurements after outgassing at rt under vacuum.

### Description of the Samples

Three systems were prepared under different combinations of treatments as follows: (a) G_TF_/AX_160_: (i) dehydration of silica at 160 °C for 2 h under vacuum; (ii) 8 cycles of CVD with TF between rt and 160 °C (corresponding to a total time of 20 h Gly sublimation). G_TF_/AX_rt_ was also prepared for the sake of comparison, using the same procedure but with a dehydration at rt instead of 160 °C before CVD. (b) G_TFHF_/AX_160_: (i) dehydration of silica at 160 °C for 2 h under vacuum; (ii) 3 cycles of CVD with TF between rt and 160 °C; (iii) several cycles of HF; (iv) 5 cycles of CVD with TF between rt and 160 °C (corresponding to a total time of 20 h Gly sublimation). In other words, this sample differed from G_TF_/AX_160_ by the insertion of HF cycles between the third and fourth steps of Gly CVD. (c) G_TFHF_/AX_WD_: (i) WD cycles of the silica support; (ii) 8 cycles of CVD with TF between rt and 160 °C; (iii) several cycles of HF; (iv) several cycles of WD; and (v) 6 cycles of CVD with TF between rt and 160 °C (corresponding to 35 h Gly sublimation in total). All samples were subjected to HF cycles followed by H/D exchange at the end of the polymerization reaction.

### Infrared (IR) Spectroscopy

IR spectra were recorded using a Bruker Vector 22 instrument with a DTGS detector at beam temperature (bt) (ca. 50 °C) in a spectral window of 400–4000 cm^–1^ using a resolution of 4 cm^–1^ and accumulating 64 scans to have a good signal-to-noise ratio. For the IR measurements, the powder of pristine silica (or of samples obtained after the thermal treatment) was pressed into self-supporting pellets and placed in a traditional IR cell with CaF_2_ windows, equipped with a valve to be connected to a vacuum line of a residual pressure of ca. 1.0 × 10^–5^ mbar where all experiments of adsorption/desorption were performed in situ.

### X-ray Diffraction (XRD)

X-ray powder diffraction patterns for the samples were recorded on a PANalytical X’Pert diffractometer using a Cu Kα (λ = 1.5405 Å) radiation source and working at 30 mA and 40 kV. The diffractograms were recorded with a scanning range set between 10° and 45° 2θ, a step size of 0.01° 2θ, and a dwell time of 1 s per step.

### High-Resolution Mass Spectrometry (HR-MS)

The supernatant obtained from each washing suspension was removed and used for analysis by high-resolution mass spectrometry using an LTQ Orbitrap mass spectrometer (Thermo Scientific) equipped with an atmospheric pressure interface and an electrospray ionization (ESI) source in negative ion mode. The source voltage was set to 4.48 kV. The heated capillary temperature was maintained at 270 K. The mass accuracy of recorded ions (vs calculated) was ±1 mmu (without internal calibration). The samples were delivered directly to the mass spectrometer via a Hamilton microliter syringe at a constant flow (10 μL/min). Data acquisition and processing were performed using the Xcalibur software.

### Peak Fitting

The IR spectra were analyzed using OriginPro 2018 (OriginLab Corporation, Northampton, MA). Nonlinear fitting of the peaks in the spectral data was performed using a peak analyzer (adopting the Levenberg–Marquardt algorithm). Baseline corrections were executed using a second derivative (zeroes) method to find anchor points and determine the baseline. Hidden peaks were specified using a second-derivative method followed by smoothing with the 30–40 points Savitsky–Golay function of a polynomial order of 2. The peak fitting was then performed using the Gaussian function
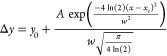
1where *y*_0_ is the offset, *A* the area, *x*_c_ the center, and *W* the Gaussian full width at half-maxima (FWHM). The baseline, peak center, and peak width parameters were fixed and released during fitting to help initializing the parameters. The iteration procedure was stopped when the best fit was reached (reduced χ^2^ < 1 × 10^–9^). The secondary structures contents are reported as integrated areas of the corresponding fitted bands. Their percentage was evaluated by dividing the areas assigned to a specific secondary structure by the total area under the amide I band (most commonly used band to study the secondary structure of peptides, 1600–1700 cm^–1^). The assignments of the various components were made using the ranges corresponding to structural elements as reported in Barth’s review.^18^ Note that the β-sheet structure gives rise to two components in separate ranges due to excitonic splitting and that amide I deconvolution is facilitated in the case of polyglycine by the absence of absorption due to side chain groups.^19^

## Results and Discussion

Amorphous silica of the AEROSIL type has been used in several works dealing with the abiotic polymerization of amino acids.^4,20,21^ In our study, we selected the amorphous AEROSIL OX 50 (AX) silica, characterized by a specific surface area (SSA) of ca. 50 m^2^ g^–1^, high enough to get clearly detectable IR signals of the surface species, which had been previously demonstrated to cause the formation of linear oligopeptides from amino acids.^4^

### Difference in the Polymerization Reaction between a System Subjected to Temperature Fluctuations and Another One Subjected to Both Temperature and Humidity Fluctuations

**Figure 1:**
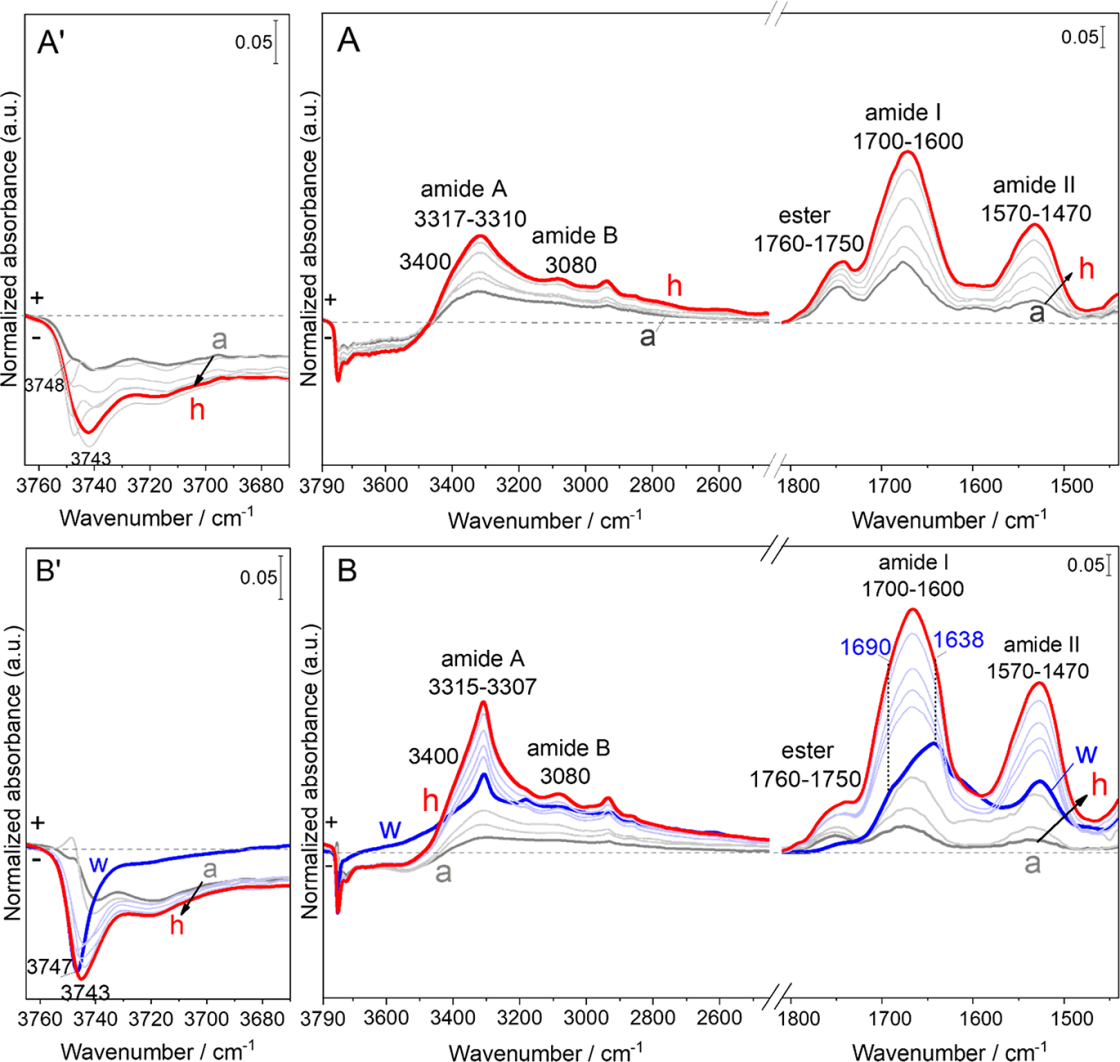
IR difference spectra resulting from Gly sublimation at 160 °C by CVD performed from 2.5 h (a) to 20 h (h) on the two samples: (A) G_TF_/AX_160_, Gly adsorbed on silica pretreated at 160 °C, and (B) G_TFHF_/AX_160_, Gly adsorbed on silica pretreated at 160 °C and subjected to intermediate HF cycles during CVD. Panels A′ and B′ show a magnification of the corresponding spectra in the silanols’ OH stretching region.

After dehydration of the silica at 160 °C under vacuum for 2 h, two samples were prepared with different procedures as explained in the Experimental Section: one subjected to temperature fluctuations (TF) during the adsorption of Gly monomers on the surface by CVD and the other one to both TF and hydration fluctuations (HF) cycles. The samples are labeled as G_TF_/AX_160_ and G_TFHF_/AX_160_, respectively. The IR difference spectra of G_TF_/AX_160_ and G_TFHF_/AX_160_ samples during the successive cycles of the 20 h CVD are presented in Figures 1A and 1B, respectively. In panels A′ and B′, the intensities in the silanol’s OH stretching region are enhanced for the sake of clarity. Gray curves show intermediate sublimation steps of 2.5 h. Light blue curves show intermediate CVD steps of 2.5 h following the HF cycle (bold blue curve (w)). The corresponding spectrum of the material obtained before the start of CVD process (AX_160_) is subtracted as a baseline.

From the first CVD cycle, the amide I (1700–1600 cm^–1^) and amide II (1570–1470 cm^–1^) bands are observed, corresponding to ν_CO_/δ_NH_ and δ_NH_/ν_CN_ vibrations, respectively. The high intensity ratio of amide II to amide I is an indication of the formation of linear peptide chains instead of cyclic ones, while the band at 1760–1750 cm^–1^ may be assigned to ester groups^22^ formed between the peptide chains and surface silanols.^12^ Each step of glycine CVD causes an increase of the ester groups (positive signal in the difference spectra of Figure 1A,B) together with a decrease of the nearly free silanols (NFS) associated with the band at 3743 cm^–1 ^^23^ (negative signal in the difference spectra of Figures 1A′ and 1B′): both ester and NFS represent crucial elements for monomers activation and polymerization.^12^ Further confirmation for the formation of linear peptides can be found in the amide A (3315–3307 cm^–1^) and B (3080 cm^–1^) bands that arise from the ν_NH_ in the peptide units. In this sample, the amide A signal is broad and probably composite.

**Figure 2:**
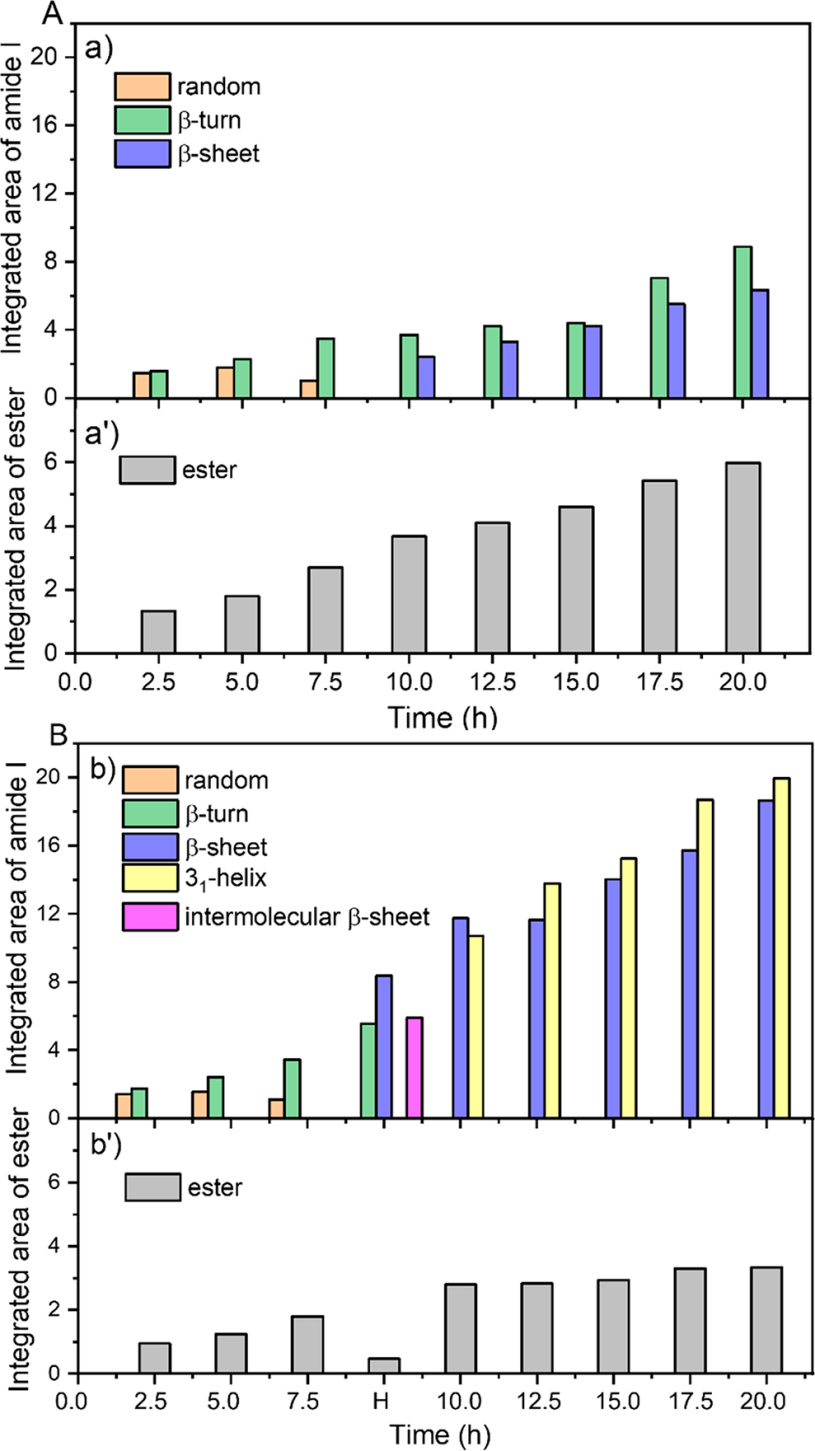
Evolution of (a) and (b) the absolute intensities of the different types of secondary structures and (a′) and (b′) integrated area of ester groups as a function of time during Gly deposition by CVD during the different cycles on the two samples: (A) G_TF_/AX_160_ and (B) G_TFHF_/AX_160_.

Both treatments illustrated in Figure 1A,B start with three cycles of glycine CVD, and indeed the spectra up to that point are very similar, confirming the reproducibility of the experiment. After the first three CVD cycles, G_TFHF_/AX_160_ was subjected to HF cycles, resulting in spectrum (w) in Figure 1B, recorded after outgassing under vacuum, which shows significant changes in the ν_OH_ pattern and in the shape of the peptide bands. For the ν_OH_ region (Figure 1B′), the admission/outgas of the water vapor results in modifications of the negative band in the silanol groups region, which indicates that the interaction of the silica surface with water vapor changed the Si–OH population. More specifically, NFS consumed during the first CVD steps are restored (the signal at 3743 cm^–1^ becomes less negative), and isolated silanols are removed (sharp negative signal at 3747 cm^–1^). With regard to the peptide bands, an important change in the shape and intensity of the amide I band is seen with the appearance of separate components at 1638 and 1690 cm^–1^, associated with the formation of β-sheet conformations^24^ (more discussion on this matter in Figure 2). Moreover, a significant narrowing of the amide A band occurs along with a further increase in the intensity of the amide II band. This is coupled with a strong decrease in the intensity of the ester band, although it does not disappear completely (Figure 2B, b′). When glycine CVD resumes, the amide I and II bands for G_TFHF_/AX_160_ exhibit an important and abrupt increase with respect to the sample G_TF_/AX_160_ (see Figure S1). Later on, the amide I intensity increases at the same rate in both samples. The narrow amide A component keeps growing during the subsequent steps for G_TFHF_/AX_160_ while a broader component becomes apparent at higher wavenumbers (around 3400 cm^–1^). While the band associated with the ester groups (1760–1750 cm^–1^), which represent the anchors of the peptide chains to the silica surface, undergoes a continuous increase in intensity along the 20 h CVD for G_TF_/AX_160_ (Figure 2A, a′), resuming CVD after the HF cycles (G_TFHF_/AX_160_) abruptly restores it to a rather high intensity, after which only a negligible increase is observed (Figure 2B, b′).

All of these observations can be explained by the following scenario: glycine CVD causes both an increase in the length of already existing peptide chains by monomer condensation and the appearance of new chains by ester formation with NFS centers.^12^ Ester formation is normally slow because access of the Gly monomers to the surface is partly hindered by the growing peptide chains. When water vapor is admitted, some of the ester links are hydrolyzed and the corresponding peptide chains are detached from the surface. They become partly mobile and able to rearrange, forming regularly H-bonded aggregates with other chains. These highly self-organized aggregates are characterized by the narrow amide A band and a specific signature in the amide I and II regions (see below). When glycine is readmitted, newly freed, NFS-rich regions on the surface quickly react with glycine monomers to form nucleating sites for additional chains, following which both aggregated and newly formed chains start growing again. More specifically, we believe that these two types of chains correspond to the two components in the amide A region: well-organized, regularly H-bonded chains to the narrow band at 3300 cm^–1^ as already mentioned and more disordered, weakly H-bonded chains to the broader band around 3400 cm^–1^. This correlation of the amide A position with the H-bonding state is in line with previous observations^25^ and theoretical calculations.^26^ The different integrated areas are obtained as a result of a peak fitting done on the ester (1760–1750 cm^–1^) and amide I (1700–1600 cm^–1^) bands. The amide I band is very sensitive to the secondary structure of peptides and often used to identify it. Therefore, for each CVD cycle, we identified, separated, and quantified its components using an enhanced peak fitting method based on the second derivative of the IR profiles. The absolute intensities of each secondary structural components are reported for both G_TF_/AX_160_ and G_TFHF_/AX_160_ in Figures 2A, a and 2B, b, respectively. The relative contents of the different secondary structures (see the Experimental Section) have also been evaluated as percentage values for both samples. The integrated areas of the ester bands, already discussed above, are also reported as a function of the CVD time (Figures 2A, a′ and 2B, b′).

The quantitative analysis of the secondary structures shows the fast initial formation of random and β-turn conformations, representing about 45% and 55%, respectively, of the total amide I band after the first CVD cycle. For the following two CVD cycles (up to 7.5 h CVD), the amount of β-turn conformations increases progressively (up to 77%) at the expense of the random structures. For G_TF_/AX_160_ (Figure 2A, a), a significant transition is observed after 10 h CVD, where the polyGly chains start forming more ordered structures, consisting of β-sheets, while random chains have disappeared. The relative proportions of β-sheets and β-turns vary little until the end of the 20 h CVD, where the proportion is 42% to 58%.

On G_TFHF_/AX_160_, the distribution of secondary structures is similar to G_TF_/AX_160_ for the first three cycles of CVD (Figure 2B, b), confirming the reproducibility of the observations. The HF cycles applied after that promote the significant formation of ordered structures, including 42% β-sheet and 30% intermolecular β-sheet while random coils disappear and β-turn conformations fall down to only 28% of the total band area (Figure 2B). This is the stage where the integrated area of the ester band exhibits a significant decrease.

The following five CVD cycles show a mixture of ordered structures containing both β-sheets and 3_1_-helices as polyglycine II (PG II)^27−33^ in comparable amounts (49% to 51% at the end of the 20 h CVD). This is coherent with the fact that N-terminal domains are usually rich in 3_1_-helices formed due to self-assembly of peptides that results from hydration with a subsequent hydrogen bond formation.^16,34^

**Scheme 1:**
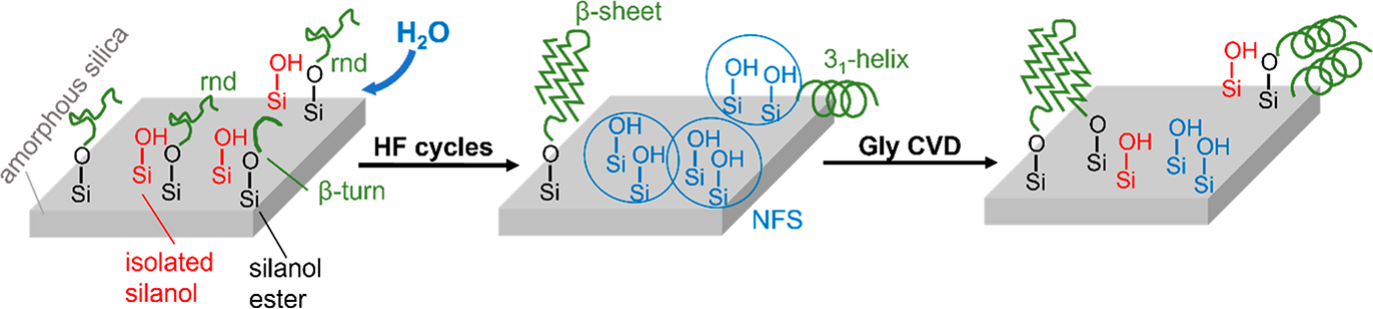
Suggested Scheme for the Polymerization of Peptides on Amorphous Silica during Gly CVD with Intermediate HF Cycles

One may wonder why 3_1_-helices are formed in high quantity after the water exposition step, while only β-sheets are encountered in the sample that did not undergo such a step. One possibility is that detached chains formed by hydrolysis may either agglomerate to existing β-sheets (whose absolute abundance indeed increases) through the formation of hydrogen bonds of N–H···O=C among neighboring chains, or rearrange independently of them to form 3_1_-helices, which would not strongly interact with the surface. When CVD resumes, however, the amounts of both types of secondary structures grow constantly, indicating that they can both impart their structure to newly formed chains. These transformations are sketchily summarized in Scheme 1.

It is important to underline that during this step preformed polyglycine chains exert a 2-fold templating influence on the newly formed ones. First, as already stated, they define the type of secondary structure that the latter will adopt. Second, judging from the absolute values of the amide I components in Figure 2, the agglomeration of new Gly monomers is faster and reaches higher final amount in G_TFHF_/AX_160_, containing 3_1_-helices and β-sheets, than in G_TF_/AX_160_. Therefore, it seems likely that gas-phase Gly monomers have a higher probability of condensing with the growing end of the corresponding chains because they are prepositioned by H-bonding, in a form of template-directed synthesis.^35^ In the origins of life field, template-directed polymerization has long been tested for growing RNA chains,^36^ but it is more surprising to find it at work for polypeptide chains. Note that if this interpretation is correct, the promoting effect on polyGly chain growth at this stage would only be indirectly due to the silica surface, as opposed to the direct implication of surface groups observed at the beginning of CVD.^12^

### Structural Dynamics of the Peptide Chains Revealed by H/D Exchange

**Figure 3:**
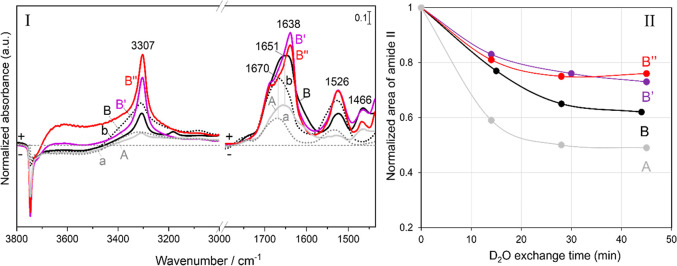
Panel I represents the IR difference spectra obtained (a and b) directly after Gly sublimation on silica for 20 h; (A and B) after subsequent H/D exchange and then outgassing of D_2_O at bt until invariance of spectra, on the two samples: G_TF_/AX_160_ and G_TFHF_/AX_160_, respectively. For G_TFHF_/AX_160_, spectra obtained after a first washing with liquid water at rt (B′) and a second washing with liquid water while heating at 70 °C (B″) on G_TFHF_/AX_160_ are also displayed. Panel II represents the evolution of the amide II band area during D_2_O adsorption/desorption, after the end of the HF cycles (corresponding to time 0 for A and B) or after the end of the WD cycles (at time 0 for B′ and B″). In each case, band intensities have been normalized to a value of 1 at time = 0.

After Gly CVD for 20 h, both G_TF_/AX_160_ and G_TFHF_/AX_160_ were exposed to HF cycles followed by D_2_O vapor exposure until invariance of in-situ IR spectra. The admission/outgassing of water vapor on both samples at this stage results in a significant change in the peptide bands of the IR signals (data not shown). These changes in amide A, amide I, and amide II bands were more clearly evidenced after subsequent D_2_O adsorption/desorption cycles until invariance of spectra (Figure 3, panel I, spectra A and B).

For both G_TF_/AX_160_ and G_TFHF_/AX_160_, after 45 min of H/D exchange (curves A and B, respectively), the amide II band exhibits a decrease in intensity while a new band appears at 1466 cm^–1^ corresponding to the amide II vibration of the deuterated peptide linkages, sometimes called amide II′ (this band is strongly displaced due to its significant NH bending component). It is noteworthy, however, that the original amide II linkages do not disappear completely, indicating that some protonated peptide links cannot be exchanged. This suggests that part of the peptide moieties is inaccessible to D_2_O diffusion.

The IR profiles after H/D exchange also exhibit a narrow amide A band (3307 cm^–1^) as compared to the spectra (a and b) collected directly after Gly sublimation by CVD; in G_TFHF_/AX_160_, that clearly contained one narrow and one broad component, and the broad component at 3400 cm^–1^ has disappeared. In line of our previous assignments, this would mean that the weakly H-bonded structures can be exchanged by D_2_O, while the strongly H-bonded ones resist exchange, as might have been intuitively expected. The deuterium-exchanged counterpart of the amide A band appears at around 2760 cm^–1^.

The amide I band (around 1670 cm^–1^) is not expected to be strongly shifted by H/D exchange, but still it undergoes a change in shape and intensity with a shift of its maximum to 1651 cm^–1^ as a result of a decoupling of vibrations due to the transformation of some N–H moieties in amide I to N–D ones.^4^

Both phenomena are more clearly marked for G_TFHF_/AX_160_ (curve B, Figure 3, panel I) as compared to G_TF_/AX_160_ (curve A, Figure 3, panel I) which reflects the higher amount of ordered self-assembled structures formed on the surface of the former. In order to study the resistance of these self-assembled structures on G_TFHF_/AX_160_ to harsher treatments, the sample was subjected to a first set of several cycles of wetting/drying (WD) with liquid water at rt followed by a second set of several cycles of wetting with liquid water at 70 °C. The IR profiles recorded after invariance of spectra at the end of each set (Figure 3, panel I as curves B′ and B″, respectively) show a further significant change in shape, increase in intensity, and appearance of a sub-band at around 1638 cm^–1^ for the amide I band. The latter is not due to adsorbed water because no strong absorbance is observed in the 3000–3500 cm^–1^ region (O–H stretching), and it probably corresponds instead to the formation of more β-sheet structures.^37^ Meanwhile, the narrow amide A band at 3307 cm^–1^ increases in intensity while the amide II band shows resistance to deuteration (smaller ratio of the deuterated to the protonated species, as compared to the sample that had not been submitted to WD cycles). The latter feature may be explained by the fact that WD cycles induce the formation of a higher amount of ordered and tightly packed aggregates^4^ that prevent the diffusion of the D_2_O molecules. These aggregates cannot be desorbed from the silica surface by washing with liquid water even at high temperature. Indeed, long oligoglycine peptides have a low water solubility:^30,38,39^ they have more affinity for other polyglycine chains than for the aqueous phase.

In order to obtain more information about the structural dynamics (flexibility and degree of solvent accessibility of the formed peptides), the kinetics of H/D exchange in peptide links was followed by monitoring the residual intensity of the amide II band as a function of the sample exposure time to D_2_O during all the intermediate cycles of adsorption/desorption. The fraction of nonexchanged residues, calculated using eq 2, is plotted as a function of D_2_O exchange time in Figure 3, panel II.^40,41^
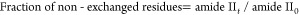
2where amide II*_t_* and amide II_0_ are the integrated areas of amide II band at time *t* and 0 of D_2_O exchange, respectively. The amide H/D exchange rate is faster in G_TF_/AX_160_ (curve A) than in G_TFHF_/AX_160_ (curve B). After the first 15 min of D_2_O adsorption/desorption cycles, around 41% of the amide groups of polyGly on G_TF_/AX_160_ were deuterated, while only 23% were exchanged in G_TFHF_/AX_160_.

**Figure 4:**
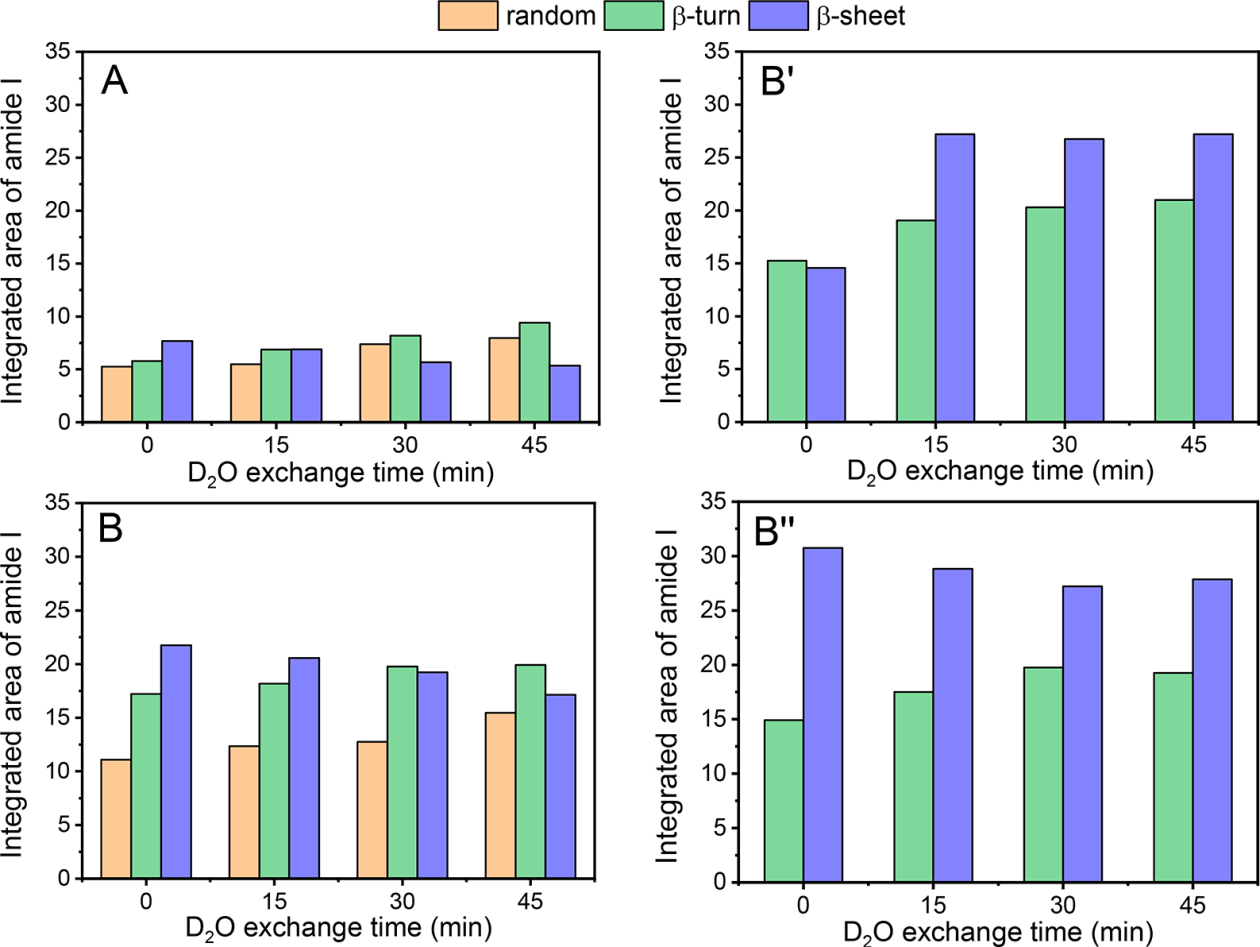
Evolution of the integrated area of the different types of secondary structures at the end of the HF cycles (time 0) and during subsequent D_2_O adsorption/desorption (time >0) on the two samples, G_TF_/AX_160_ (A) and G_TFHF_/AX_160_ (B), and after washing of the latter with liquid water at rt (B′) and at 70 °C (B″). The different integrated areas are obtained from peak fitting in the amide I band range (1700–1600 cm^–1^).

We also quantified the different types of secondary structures that evolved during the H/D exchange performed after 20 h CVD and hydration (Figure 4A,B) and after WD cycles at rt (Figure 4B′) and at 70 °C (Figure 4B″) because the kinetics of the amide H/D could be related to the rigidity of the peptide secondary structures. Quite unexpectedly, the final HF cycles caused the destruction of the 3_1_-helices that had been present after CVD in G_TFHF_/AX_160_ (52% after 20 h CVD, Figure 2B) and were replaced by less organized structures: β-turns (32%) and random coils (20%; Figure 4B, time = 0). This is not unprecedented as proteins adsorbed on solid surfaces have been observed to lose their 3_1_-helices structural elements to random coils and turns upon exposure to water.^42^ In G_TF_/AX_160_, the β-turns that represented the majority structure after CVD (58% after 20 h CVD, Figure 2A) decreased to around 31%, probably transformed into random coils (28%; Figure 4A, time = 0). The remaining β-sheets represented 41% of the total secondary structures in G_TF_/AX_160_ and 48% in G_TFHF_/AX_160_ (Figure 4A,B, time = 0). Altogether, after the HF cycles, the proportions of different secondary structure components were similar in G_TF_/AX_160_ and G_TFHF_/AX_160_, although the global intensity of the amide bands was higher in the latter. It might have been expected that the exchangeable fraction of peptide chains corresponds to the β-turn and random conformations since they present disordered, shorter, and/or more flexible structures that should allow easier D_2_O diffusion. At the end of the H/D exchange treatments, the exchanged percentage of peptide chains reached 52% for G_TF_/AX_160_ and 38% on G_TFHF_/AX_160_ (Figure 3, panel II), as compared to 76% and 67% respectively for the β-turn + random coils (Figures 4A and 4B, respectively, at time = 45 min). The trend is the same for the two observables, but the exchanged amounts remain smaller than the disordered configurations contents. Possibly, even some of the disordered structures are not quickly exchangeable, but precise quantification could also be complicated by differences in the extinction coefficients. Further complication is that during D_2_O exchange (Figure 4A,B, time >0) the amount of β-sheet slightly decreased while those of β-turns and random coils increased for both samples. D_2_O vapor, like H_2_O vapor in the HF cycles, seems to turn ordered into disordered structures, but the kinetics of this disordering is slow. G_TFHF_/AX_160_ was submitted to WD cycles at rt and while heating at 70 °C. A conspicuous effect of these washing treatments is the disappearance of the random coil components in the deconvolution of the amide I (Figure 4B,B′). Most likely, these correspond to rather short and therefore more soluble chains that may be eliminated by washing. The peptide chains remaining on the surface are well-ordered, strongly H-bonded aggregates, consisting in a majority of β-sheets (around 60% of the integrated amide I area, Figures 4B′ and B″), as was already apparent from the discussion of Figure 3, and therefore the amount of H/D exchange is limited (25% after 45 min; Figure 3, panel II, curves B′ and B″, respectively). In addition, the exchange kinetics is slower with respect to what is observed before WD cycles (Figure 3, panel II, curves A and B).

### Effect of HF and WD Cycles on Gly Deposition for Extended Durations

In the following part, we study the deposition of glycine on strongly hydrated silica surfaces. It is known that the nature and local arrangements of the silica surface groups (silanols and siloxane rings) may induce either a hydrophobic or a hydrophilic behavior, respectively, characterized by a heat of water adsorption lower or higher than the latent enthalpy of liquefaction (44 kJ mol^–1^).^43^ In fact, it has been found that the surface density of silanol groups on the amorphous silica surface is simply related to the hydrophobic/hydrophilic character: a silica surface with 4–5 Si–OH/nm^2^ is hydrophilic because water molecules adsorb through strong H-bonding to silanol sites, while a surface with 1–2 Si–OH/nm^2^ is hydrophobic because most water molecules interact with the siloxane bridges.^44−46^ Silica AX50 has a well-defined heat of water adsorption estimated to ca. 40 kJ mol^–1^, i.e., below the latent enthalpy of liquefaction;^47^ thus, it may be considered as somewhat hydrophobic due to a relatively low concentration of silanol groups. This native silica, after outgassing at rt, is labeled as AX_rt_. In parallel, another sample was prepared where several WD cycles were applied to the silica support, also followed by an outgassing under vacuum at rt (labeled hereafter as AX_WD_). The corresponding IR profile obtained, compared to that of AX_rt_ in Figure S2, showed a strong decrease of the band at 3747 cm^–1^ accompanied by a significant positive broad band centered at 3450 cm^–1^, which indicates the transformation of the isolated silanols to H-bonded ones due to their interaction with adsorbed water. This was coupled with the appearance of an intense narrow band peaked at 3742 cm^–1^ which corresponds to the formation of nearly free silanols (NFS). Indeed, several studies have demonstrated that the contact of silica with H_2_O at room temperature can result in the fast opening of the highly strained siloxane bridges such as (SiO)_2–3_ to give two vicinal (weakly H-bonded) silanol sites.^48−50^ Thus, AX_WD_ constituted an appropriate sample to study the effect of NFS on the polymerization reaction.

The IR profiles collected after successive steps of Gly sublimation for a total of 20 h CVD (Figures S3 and 5a–h) show that G_TF_/AX_WD_ exhibits higher intensities of the characteristic bands of linear peptides (amide I, amide II) as compared to G_TF_/AX_rt_. The amide A band is also more intense, with two components at 3400 cm^–1^ (originally predominant) and 3300 cm^–1^ (developing later). Furthermore, a certain number of zwitterionic Gly monomers are formed on the surface during polymerization as shown by the appearance of bands at 1595 and 1413 cm^–1^ which may be assigned to ν_as,COO_^–^ and ν_s,COO_^–^ of Gly monomers, respectively^21^ —these bands were not present in the previously discussed samples that had been pretreated at 160 °C, and therefore the stabilization of zwitterionic glycine is probably correlated to the presence of adsorbed water.^51^ In the silanols region (inset of Figure S3), the NFS groups were selectively removed during Gly deposition on G_TF_/AX_WD_ (negative band at 3742 cm^–1^), while this removal is accompanied by the formation of new isolated silanols due to the condensation of some siloxane rings on G_TF_/AX_rt_ upon heating at 160 °C during CVD (positive band at 3747 cm^–1^ for isolated silanols in parallel with a negative band at 3742 cm^–1^ for NFS). This reflects that G_TF_/AX_WD_, with more NFS which constitute essential elements for monomers activation and polymerization,^12^ represents a more efficient platform for the formation of polyGly chains.

**Figure 5:**
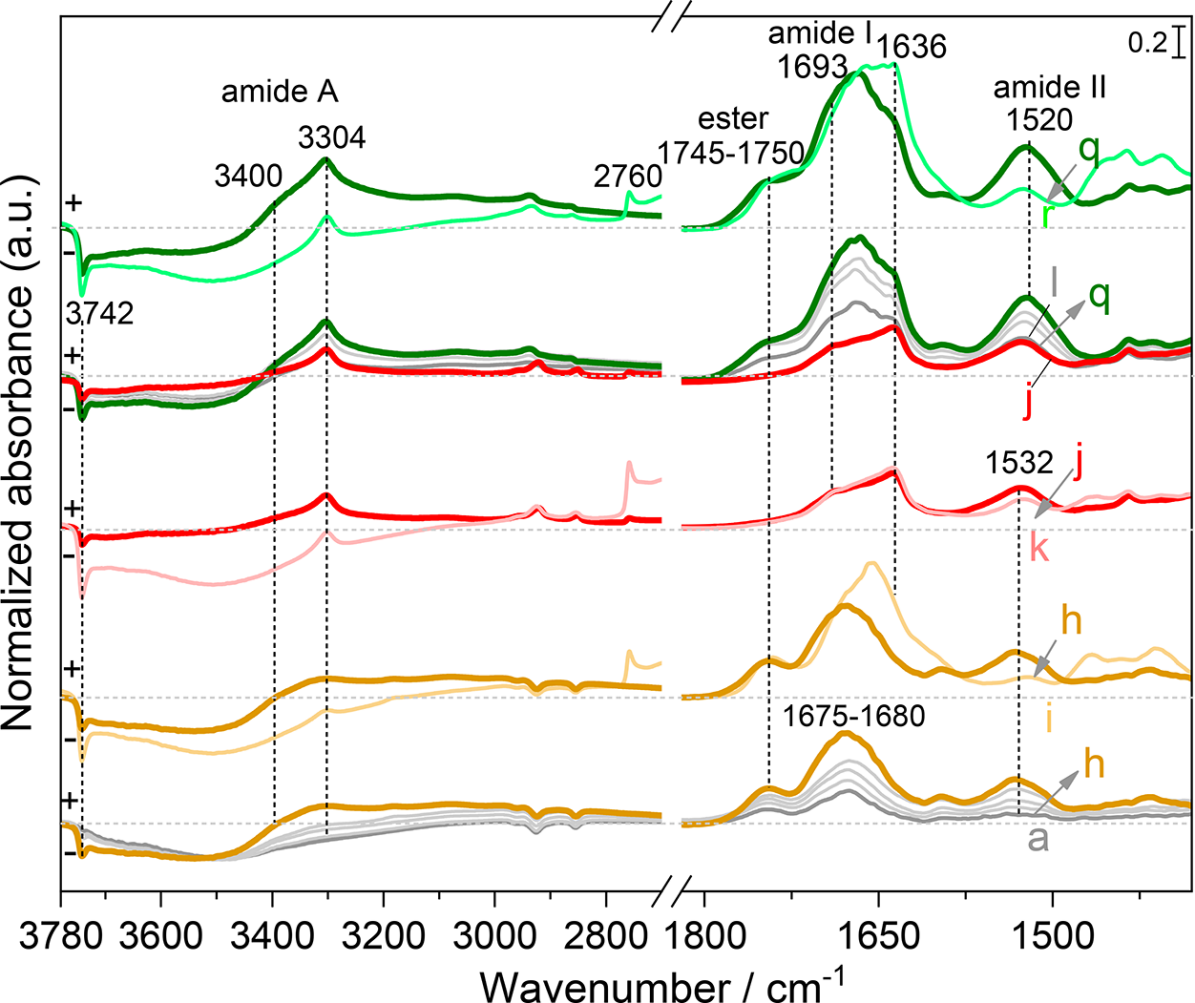
IR difference spectra on G_TFHF_/AX_WD_ resulting from Gly sublimation at 160 °C by CVD with TF cycles measured from 2.5 h (a) to 20 h (h); (i) after D_2_O adsorption/desorption cycles at bt until spectral invariance; (j) after wetting/drying cycles with liquid water and outgassing at rt; (k) after subsequent D_2_O adsorption/desorption cycles at bt; a second set of ^15^N-Gly sublimation at 160 °C with TF cycles, by CVD measured from 2.5 h (l) to 15 h (q); (r) after D_2_O adsorption/desorption at bt until spectral invariance. Gray curves show intermediate sublimation steps of 2.5 h. For all spectra, the spectrum of the silica support before the start of CVD process (AX_WD_) has been subtracted as a baseline.

After the first 20 h sublimation under TF cycles, the G_TF_/AX_WD_ sample was subjected to HF cycles followed by H/D exchange until invariance of spectra. At this stage, the sample is designated as G_TFHF_/AX_WD_. The spectrum (Figure 5, curve i) shows a change in shape and intensity of the amide I along with a decrease in intensity of the amide II band that is, however, not completely suppressed by the H/D exchange as well as a narrowing of the amide A band (disappearance of the broad component at 3400 cm^–1^) together with the appearance of the D-exchanged counterpart at 2760 cm^–1^. These phenomena are similar to those observed previously on G_TF_/AX_160_ (Figure 3), which were rationalized by selective D exchange of the weakly H-bonded, disordered chains leaving the strongly H-bonded agglomerates in the protonated form.

Subsequently, the sample was subjected to WD cycles followed by an outgassing at rt (Figure 5, curve j) and another H/D exchange until invariance of the spectrum (Figure 5, curve k). The IR profile at this stage shows a significant change in the shape of amide I which is now dominated by sub-bands centered at 1693 and 1636 cm^–1^, characteristic of β-sheets. The narrowing of the amide A band and the resistance of the amide II band to H/D exchange confirm that these β-sheet secondary structures are now preponderant. The XRD pattern recorded at this point (Figure S4, curve a) shows that no crystalline Gly are present on the surface. In fact, the bands at 1595 and 1413 cm^–1^ which we had assigned to monomeric glycine are no longer detectable at this stage (Figure 5, curve j), suggesting that most monomeric glycine has been desorbed by the WD treatment. The analysis by mass spectrometry of the supernatant collected after several washings of G_TFHF_/AX_WD_ indeed reveals that only Gly monomers (98%) and Gly-Gly and DKP dimers (2%) were desorbed from the surface upon washing while the polyGly resist the WD cycles and remain on the surface.

To study whether these self-assembled aggregates and the silica support remain active to promote further polymerization and elongation of the polyGly chains, two further sets of Gly sublimation by CVD with TF cycles were applied for 15 h after the WD treatment on different G_TFHF_/AX_WD_ pellets, using isotopically labeled glycine (^15^N in Figure 5 or ^13^C-Gly in Figure S5). Isotope labeling is a useful tool for vibrational spectroscopy analysis: it does not only facilitate bands assignment but also make it possible to probe specific local structures and dynamics, giving information about the mechanism of peptide aggregation and folding.^13,40,52−54^ Substituting specific atoms in a molecule by isotopes of low natural abundance results in altering the vibrational frequencies of moieties that involve this atom, without changing their chemical properties. The expected isotopic shift Δν is calculated using the equation
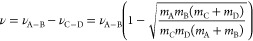
3where *m*_A_, *m*_B_, *m*_C_, and *m*_D_ are the reduced masses of atoms A, B, C, and D.^13^ Uniform ^15^N labeling downshifts the amide II by around 14 cm^–1^, while the amide A band, which arises from the ν_NH_ in the peptide units, is downshifted by ca. 8 cm^–1^ and the amide I by only ca. 1 cm^–1^. Experimental observations for the amide I are in agreement with these weak displacements.^55^

The IR profiles of G_TFHF_/AX_WD_ collected during the second set of Gly CVD (using ^15^N-Gly with TF cycles; Figure 5, curves l to q) show a further increase in the intensity of amide I and amide II bands with a downshift of around 12 cm^–1^ for the latter. This is in line with the progressive formation and prolongation of ^15^N-labeled linear polyGly chains on the surface. The amide II band must be the sum of components corresponding to ^14^N- and ^15^N-containing peptide regions, which are difficult to discriminate due to their small separation.

The amide A clearly shows the two components at 3300 and 3400 cm^–1^, both increasing in intensity along the deposition. The ester band exhibits an increase for the first 5 h CVD and then tends to a plateau. Because the ester band intensity is indicative of the number of covalent anchoring points for the polyglycine chains, it would mean that new anchored chains on the surface form only at the beginning of deposition, probably on the surface liberated by the WD treatment, while after 5 h, polymerization proceeds through the ligation of additional monomers to already existing chains. Further HF cycles followed by H/D exchange until invariance of spectra (Figure 5, curve r) reveal again a resistance to exchange of the polyGly chains in self-assembled structures on the surface (preservation of the narrow component of the amide A along with a resistance of a part of the amide II band and appearance of narrow sub-band at 1636 cm^–1^ for amide I), while the disordered structures are fully deuterated (disappearance of the broad amide A component at 3400 cm^–1^).

A different isotopic enrichment experiment was performed on a separately synthesized G_TFHF_/AX_WD_ pellet, using glycine isotopically labeled with ^13^C, instead of ^15^N, for the second set of TF (after a first set of TF and HF cycles using ^12^C Gly, followed by WD). The main interest of this experiment is to discriminate between the sub-bands of the secondary structures already formed during the first set of TF cycles and remaining on the surface after washing (which therefore contain ^12^C) and the ones of newly formed secondary structures during the second set, where ^13^C labeling downshifts the amide I band components by ca. 38 cm^–1^. Curve fitting of the IR spectra collected during the second set of CVD of ^13^C-Gly in TF cycles (Figure S5, integrated areas as a function of time) reveals that after the WD cycles the polyGly that remain chemisorbed on the surface contain 56% ^12^C β-sheet and 44% ^12^C β-turns. When proceeding with a second set of TF cycles, the first 2.5 h results in a transformation of the ^12^C β-turn into ^12^C β-sheet structures, which increase in concentration. In parallel, new ^13^C polyGly β-sheet structures are formed on the surface at the exclusion of other secondary structures. Their concentration further increases with CVD time while the ^12^C β-sheet content remains constant, as would be expected.

The integrated intensities of the ester bands are also plotted in Figure S5. The ^12^C ester groups that had resisted WD cycles remain unaffected during the subsequent CVD steps: they constitute the anchors of the self-assembled structures that have not been removed by the WD treatments. However, another band develops immediately upon CVD resumption that can be assigned to newly anchored chains, containing Si–O–^13^CO– links. These new ester links form on regions of the surface previously liberated by the WD treatment, as outlined in Scheme 1. This means that a significant part of the newly formed ^13^C-marked β-sheets belong to freshly nucleated chains. Yet these do not pass through an intermediate, disordered state as in the first set of CVD steps. The surface density of ^12^C β-sheet templates must be sufficient to impose immediate structuring of the additional chains.

## Conclusion

Systems consisting of silica, glycine in the gas phase, and water were subjected to cyclic variations in temperature and water activity to study the effect of various experimental scenarios on peptide elongation and structuring. A fluctuating system subjected to both temperature fluctuations (TF) and hydration fluctuation (HF) cycles represents a more favorable geochemical setting for the polymerization reaction as compared to a system subjected only to TF. The dehydration steps are necessary to thermodynamically drive the condensation–polymerization reaction between glycine monomers and pre-existing polyglycine chains. The role of the hydration steps is more complex. Exposure to water appears to hydrolyze some of the surface ester links that bind polyglycine chains to the silica surface, re-establishing the corresponding NFS (nearly free silanols) anchoring sites. At the same time, the freed chains partly aggregate to pre-existing polyglycine nuclei with a β-sheet secondary structure and partly form 3_1_-helices, probably weakly adsorbed. Further glycine deposition steps at low water activity cause the growth of these aggregates and the formation of new chains, with a higher amount of peptides and a higher level of structuring than in a sample not submitted to hydration. This may constitute an element of justification for the efficiency of often applied wetting-and-drying cycles. However, we are dealing with systems of much higher complexity than would be expected. The effect of water exposure will differ according to the state of the surface and growing chains, which depends on the succession of treatments applied: thus, if exposure to water happens before the beginning of CVD, it promotes the temporary stabilization of glycine monomers as zwitterions. We will deal in a later publication with the effect of initial surface state and different glycine deposition procedures on its reactivity.

The structural dynamics of the oligopeptide chains was also studied by H/D exchange and the application of washing steps. The β-sheet’s structural elements proved to be very resilient: they were inaccessible to deuterium exchange, and they were not desorbed and hardly perturbed even by liquid water at high temperature, while other structural elements with weaker H-bonds could be exchanged by D_2_O in a few tens of minutes at room temperature. These conclusions were comforted by experiments with isotopically enriched glycine deposition. It is interesting to notice that the applied experimental approach is complementary to the mass spectrometry analysis of products detached from the surface of the catalysts, which often shows the formation of DKP, Gly monomers, and dipeptide forms. The strong interaction of the poly(Gly) aggregates coupled with their low solubility points to the importance of studying the surface bound interface environment and not relying only on measurements made in solution.

From the point of view of prebiotic chemistry, the systems investigated here go further than a polymerization of amino acids, which had been observed many times. Secondary structure elements that appear in current proteins represent a higher level of structuring, one that is indispensable for protein function. It is particularly intriguing that these systems showed some hints of templated peptide growth on β-sheets, i.e., the transmission of information from nuclei to growing chains.

It will be interesting in the future to study the formation of secondary peptide structures on silica surfaces from more complex and diverse monomers, although this will complicate the analysis of IR profiles.

## Supporting Information Available

Additional graphs about the evolution of the integrated area of amide I as a function of time during Gly deposition by CVD before and after the HF cycles on the two samples: G_TFHF_/AX_rt_ and G_TFHF_/AX_160_; IR spectra of two bare silica samples before any Gly deposition when outgassed at rt (AX_rt_) or when subjected to wetting/drying cycles then outgassed at rt (AX_WD_); IR difference spectra after Gly CVD at 160 °C for 20 h on the two samples: G_TF_/AX_rt_, Gly deposition on silica outgassed at rt and G_TF_/AX_WD_, Gly deposition on silica subjected to wetting/drying cycles then sed at rt; XRD patterns for G_TF_/AX_WD_ after (a) ^12^C-Gly deposited on AX_WD_ for 20 h by CVD then subjected to wetting/drying cycles and outgassing, and (b) after ^15^N-Gly deposited on the same sample as a subsequent set by CVD for 15 h; evolution of the integrated area of the different types of secondary structures and ester as a function of time during ^13^C-Gly deposition for 7.5 h on G_TFHF_/AX_WD_ already subjected to 20 h CVD of ^12^C-Gly then subjected to WD cycles (PDF)

## Abbreviations

AX: amorphous silica
CVD: chemical vapor deposition
Gly: glycine
HF: humidity fluctuations
HR-MS: high-resolution mass spectrometry
NFS: nearly free silanols
rt: room temperature
TF: temperature fluctuations
WD: wetting/drying.
