# Supplementary material for: Polypeptide Chain Growth Mechanisms and Secondary Structure Formation in Glycine Gas-Phase Deposition on Silica Surfaces
Source: J Phys Chem B. 2023 Jan 13;127(3):673–84. doi: 10.1021/acs.jpcb.2c07382 (PMC9884078; doi:10.1021/acs.jpcb.2c07382)
Supplement: Supplementary file 1 — jp2c07382_si_001.pdf [file jp2c07382_si_001.pdf]

Supporting Information

Polypeptide Chain Growth Mechanisms and Secondary Structure Formation in Glycine Gas-Phase Deposition on Silica Surfaces

Ola El Samrout,[§,‡] Gloria Berlier,[§,] Jean-François Lambert,[‡,*] and Gianmario Martra[§,illegible]*

[§]Department of Chemistry, University of Torino, Via P. Giuria 7, 10125 Torino, Italy

[‡]Laboratoire de Réactivité de Surface, LRS (UMR 7197 CNRS), Sorbonne Université, Place Jussieu, 75005 Paris, France

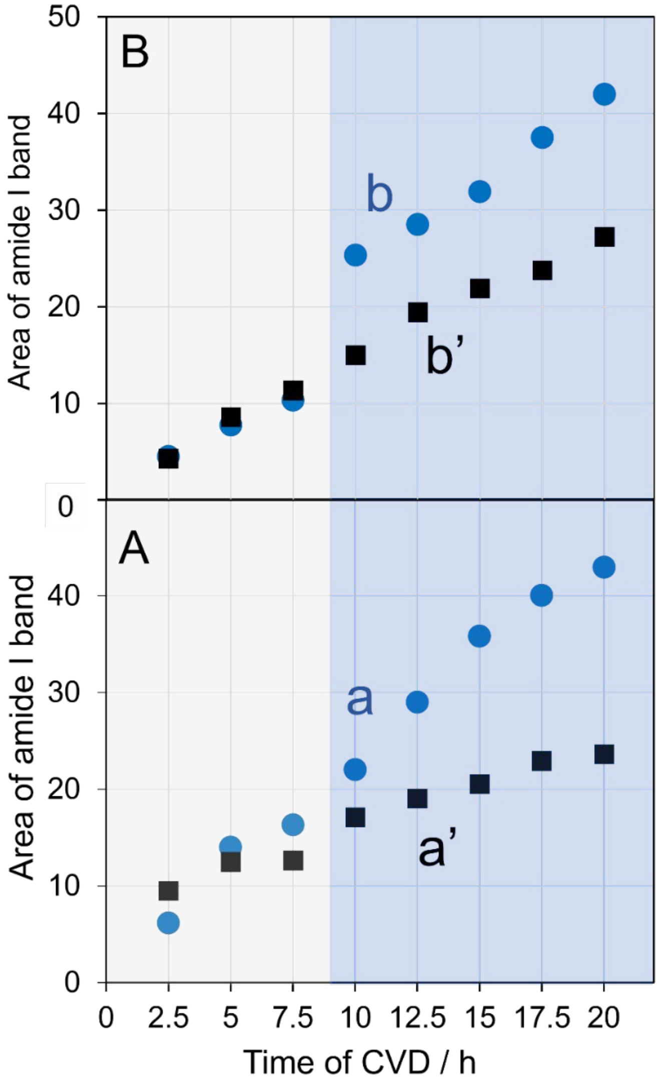

Figure S1. Evolution of the integrated area of amide I as function of time during Gly deposition by CVD before (light grey shadow) and after (blue shadow) the HF cycles on the two samples: a) G_{TFHF}/AX_{rt} and b) G_{TFHF}/AX_{160}.

Both a’) G_{TF}/AX_{rt} and b’) G_{TF}/AX_{160}, not subjected to any intermediate HF cycles, were prepared and presented for the sake of comparison.

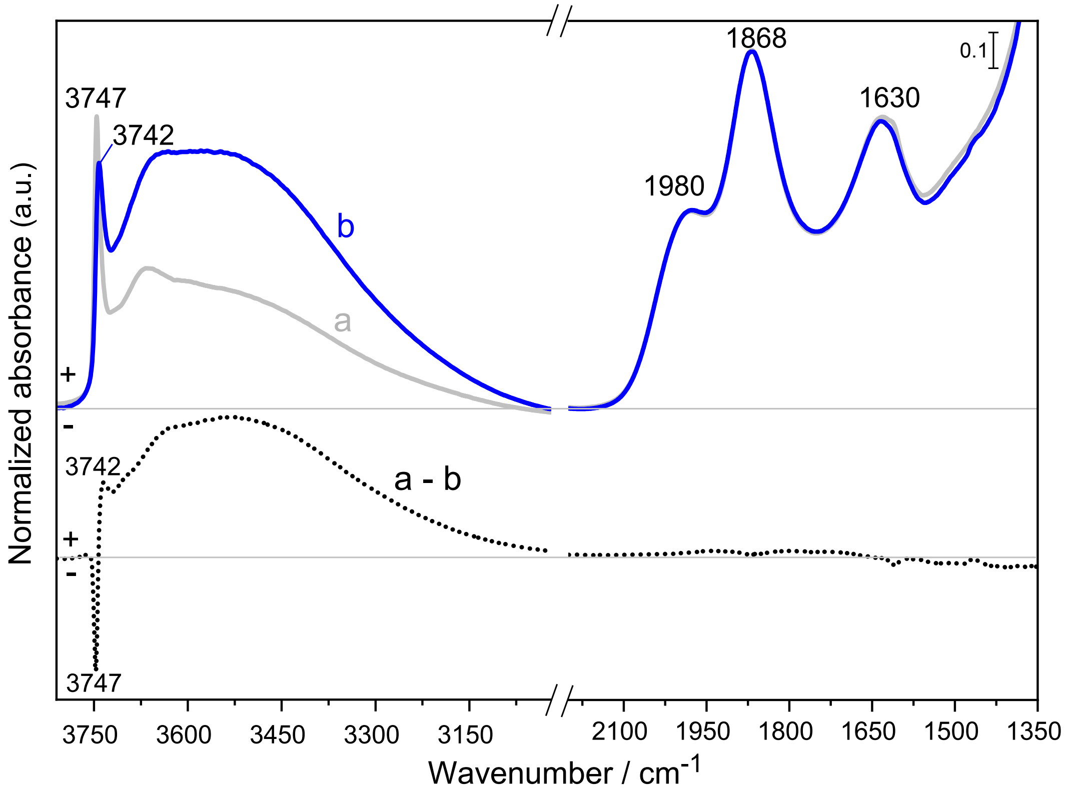

Figure S2. IR spectra of the two bare silica samples before any Gly deposition: (a) AX_{rt}, bare silica outgassed at rt and (b) AX_{WD}, bare silica subjected to wetting/drying cycles then outgassed at rt. The difference of these IR spectra (b-a) shows the effect of the surface washing on the silanol groups.

The intensity of the spectra has been normalized with respect to the optical thickness ($mg.cm^{-2}$) of the self-supporting pellets prepared for the measurements using the pattern in the 2100-1800 cm^{-1} range.

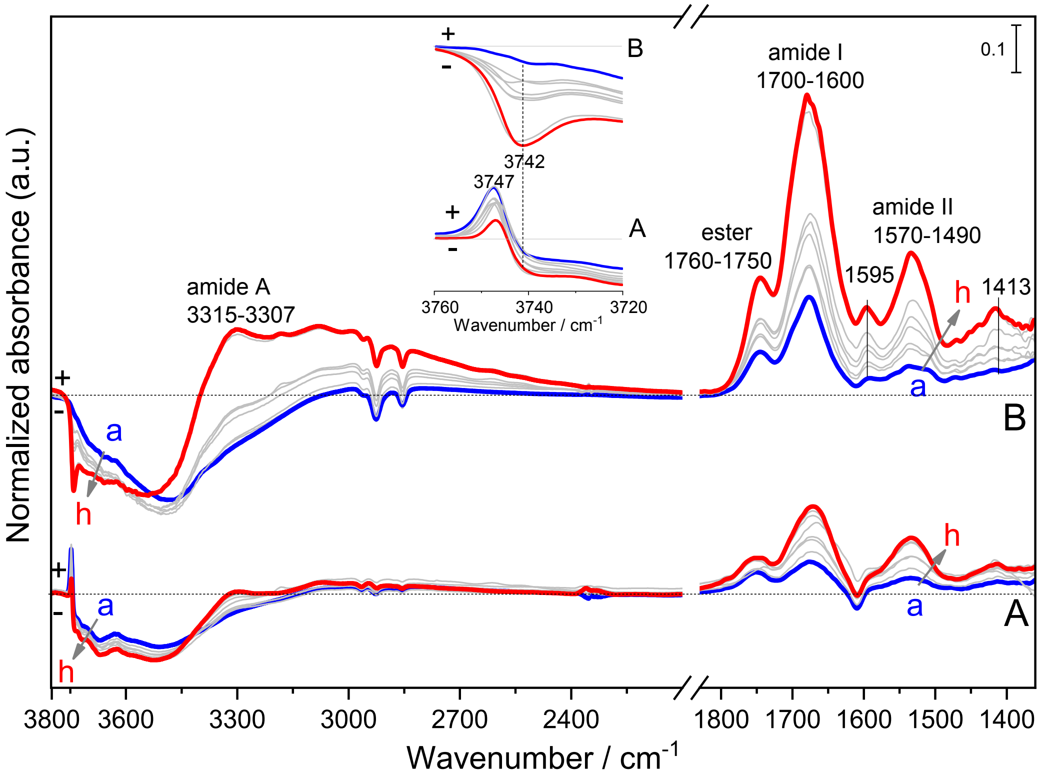

Figure S3. IR difference spectra after Gly CVD at 160 °C from 2.5 h (a) to 20 h (h) on the two samples: A) G_{TF}/AX_{rt}, Gly deposition on silica outgassed at rt and B) G_{TF}/AX_{WD}, Gly deposition on silica subjected to wetting/drying cycles then outgassed at rt. The gray curves show intermediate sublimation steps of 2.5 h.

The spectrum of the silica support before the start of CVD (AX_{rt} or AX_{WD}, respectively) has been subtracted as a baseline.

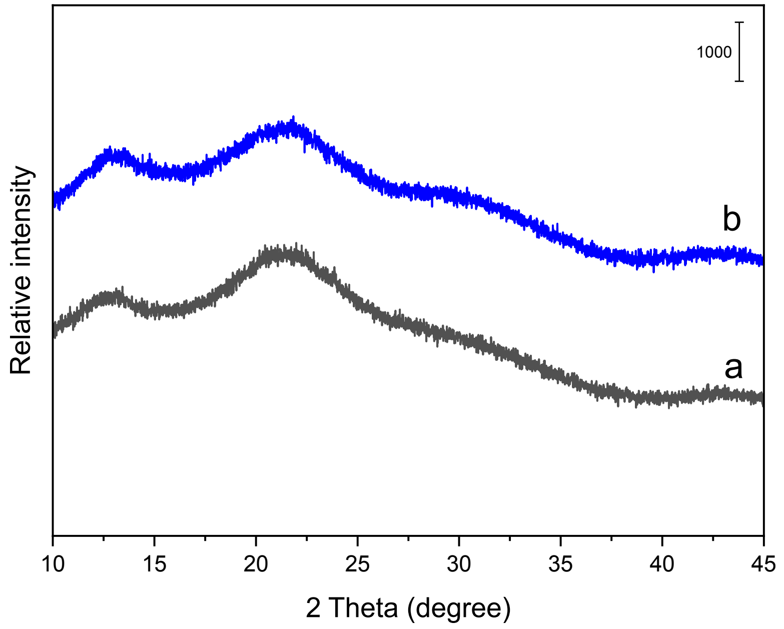

Figure S4. XRD patterns for G_{TF}/AX_{WD} after (a) ^{12}C-Gly deposited on AX_{WD} for 20 h by CVD then subjected to wetting/drying cycles and outgassed, and (b) after ^{15}N-Gly deposited on the same sample as a subsequent set by CVD for 15 h.

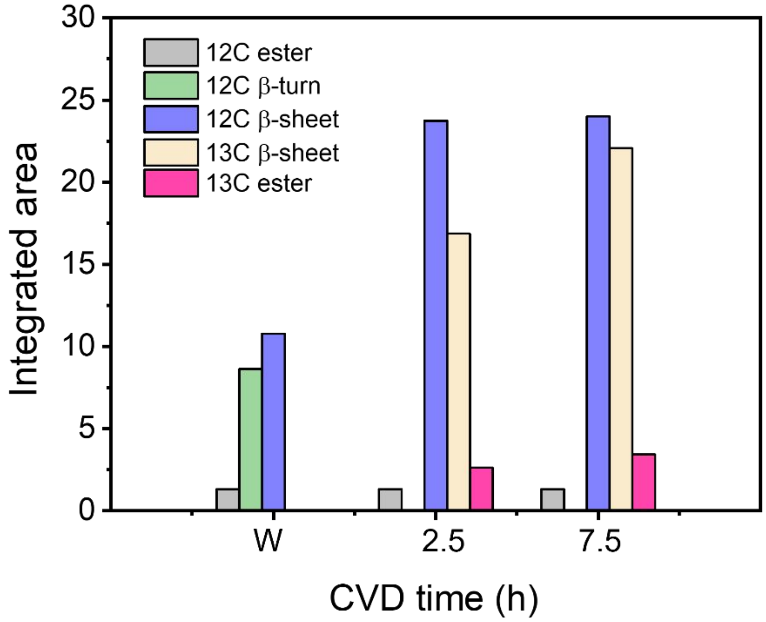

Figure S5. Evolution of the integrated area of the different types of secondary structures and ester as a function of time during ^{13}C-Gly deposition for 7.5 h on G_{TFHF}/AX_{WD} already subjected to 20 h CVD of ^{12}C-Gly then subjected to WD cycles.

The different integrated areas are obtained as a result of a peak fitting done on the ester and amide I bands of the IR spectral data.
